# Coronavirus Disease 2019 Test Results After Clinical Recovery and Hospital Discharge Among Patients in China

**DOI:** 10.1001/jamanetworkopen.2020.9759

**Published:** 2020-05-22

**Authors:** Jinru Wu, Xinyi Liu, Jianjun Liu, Han Liao, Sixuan Long, Ning Zhou, Pa Wu

**Affiliations:** 1 College of Life Science, Hunan Normal University, Changsha, China; 2 Loudi Center for Disease Control and Prevention, Loudi, China; 3 Louxing District Center for Disease Control and Prevention, Loudi, China; 4 Department of Infectious Diseases, the Second Xiangya Hospital, Central South University, Changsha, China

## Abstract

This cross-sectional study used reverse transcriptase–polymerase chain reaction tests to assess potential viral shedding among patients who previously had been diagnosed with and had clinically recovered from coronavirus disease 2019.

## Introduction

Coronavirus disease 2019 (COVID-19) spreads rapidly between cities and internationally via person-to-person transmission.^1^ Persistence of the severe acute respiratory syndrome coronavirus 2 (SARS-CoV-2) nucleic acid has been demonstrated in patients who have clinically recovered,^2^ but the overall prognosis of patients with COVID-19 after meeting the criteria for hospital discharge has not been reported, to our knowledge.

## Methods

This cross-sectional study was approved by the Hunan Normal University institutional review board, and written informed consent was obtained from all patients. This study followed the Strengthening the Reporting of Observational Studies in Epidemiology (STROBE) reporting guideline for observational studies.

After 2 discharged patients who had previously been diagnosed with and hospitalized for COVID-19 were readmitted to the hospital for symptoms of COVID-19 and found to have test results positive for SARS-CoV-2, we collected nasopharyngeal and anal swab samples from 58 other patients who had been hospitalized for COVID-19 and discharged before February 27, 2020, in Loudi, China, to evaluate potential viral persistence. For hospital discharge and in-home 2-week quarantine, previously described criteria had to be met.^2,3^

Real-time reverse transcriptase–polymerase chain reaction (RT-PCR) tests for the SARS-CoV-2 nucleic acid were performed with nasopharyngeal and anal swab samples from the discharged patients. Demographic information and laboratory findings were collected from electronic medical records.

This study used descriptive analysis. The interquartile range was calculated with Prism analysis and graphing software version 7.00 (GraphPad). Data were analyzed in February 2020.

## Results

Among the 60 discharged patients enrolled in this study, the median (interquartile range) age was 46.5 (33.5-58.5) years, and 26 (43.3%) were women. A total of 10 patients (16.7%) had RT-PCR results positive for SARS-CoV-2, including 5 patients (8.3%) with positive nasopharyngeal swab results and 6 patients (10.0%) with positive anal swab results (1 patient had positive results in both swab samples). For anonymity, these 10 patients are identified by number, as patients 1 through 10.

None of the patients with RT-PCR results positive for SARS-CoV-2 had clinical symptoms of COVID-19 after hospital readmission, except for occasional cough in patients 1 and 2, both of whom were older than 70 years with multiple underlying medical conditions. Patient 2 developed cough with sputum 5 days after hospital discharge and had RT-PCR results positive for SARS-CoV-2 on March 27, indicating a viral shedding duration of 56 days from illness onset.

Patient 4 had positive results on RT-PCR for SARS-CoV-2 with nasopharyngeal samples collected 3 weeks after hospital discharge (Figure). Patient 4 had donated plasma on February 18, 2020, to patients who were critically ill with a serum antibody (immunoglobulin G) titer of 80. Nine medical staff who collected the convalescent plasma with insufficient personal protective equipment were quarantined; however, all 9 staff had RT-PCR results negative for SARS-CoV-2 and had no symptoms in the following 2 months. No additional local cases of COVID-19 were reported after February 28, 2020.

**Figure.**
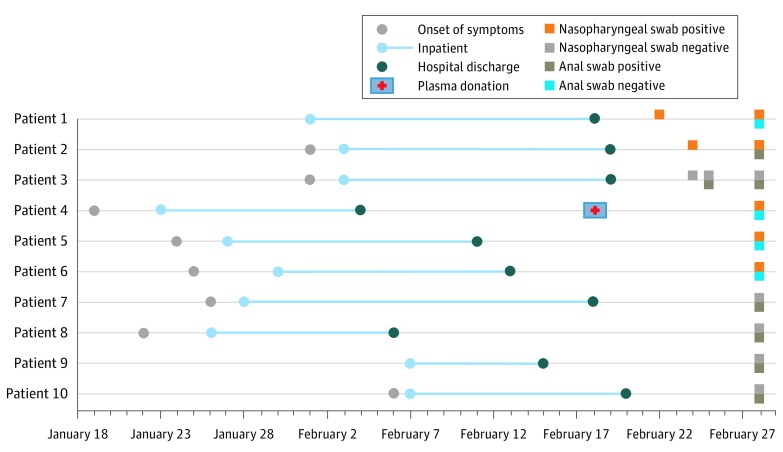
Timeline of Clinical Course of Discharged Patients With Positive Reverse Transcriptase–Polymerase Chain Reaction Test Results for Severe Acute Respiratory Syndrome Coronavirus 2

## Discussion

In this cross-sectional study, 10 of 60 patients previously diagnosed with and treated for COVID-19 had RT-PCR test results positive for SARS-CoV-2 from 4 to 24 days after index hospital discharge. As discharged patients were provided with home isolation instructions and local cases were rare, their positive results were presumed to be persistent viral shedding rather than reinfection. Consistent with previous studies showing prolonged viral shedding in the feces of patients with COVID-19,^4^ our results indicated that 6 patients had persistent viral shedding in the gastrointestinal tract after hospital discharge, including 1 patient (patient 2) who had positive results in both samples and showed RT-PCR positivity on March 27, 2020, a viral shedding duration of 56 days from illness onset. Lower threshold cycle values with anal swabs than those with nasopharyngeal swabs were identified in this study; however, the infectivity remains unclear, as infectious viruses have not been isolated from stool samples, to our knowledge.^5^

This study was limited to a small number of discharged patients who had test results positive for SARS-CoV-2. Further studies using a larger cohort and isolation of the viable virus instead of RT-PCR testing are needed to define infectivity for continued disease management after hospital discharge.

Considering the RT-PCR positivity for SARS-CoV-2 among discharged patients with COVID-19 revealed by this and a previous study,^2^ appropriate personal protective equipment for medical staff might be important while collecting convalescent plasma, and the effects of convalescent plasma from clinically recovered patients with persistent viral shedding may need to be evaluated separately.
